# PTX3 Deficiency Promotes Enhanced Accumulation and Function of CD11c^+^CD11b^+^ DCs in a Murine Model of Allergic Inflammation

**DOI:** 10.3389/fimmu.2021.641311

**Published:** 2021-07-09

**Authors:** Jyoti Balhara, Latifa Koussih, Ashfaque Mohammed, Lianyu Shan, Bouchaib Lamkhioued, Abdelilah S. Gounni

**Affiliations:** ^1^ Department of Immunology, Max-Rady College of Medicine, Rady Faculty of Health Sciences, University of Manitoba, Winnipeg, MB, Canada; ^2^ Department des Sciences Experimentales, Université de Saint-Boniface, Winnipeg, MB, Canada; ^3^ Laboratoire d’Immunologie et de Biotechnologies, EA7509-IRMAIC, Pôle-Santé, Université de Reims Champagne-Ardenne, Reims, France

**Keywords:** dendritc cells, allergic inflammation, asthma, monocytes, chemokines, allergen uptake, PTX3 protein

## Abstract

PTX3 is a unique member of the long pentraxins family and plays an indispensable role in regulating the immune system. We previously showed that PTX3 deletion aggravates allergic inflammation *via* a Th17 -dominant phenotype and enhanced CD4 T cell survival using a murine model of ovalbumin (OVA) induced allergic inflammation. In this study, we identified that upon OVA exposure, increased infiltration of CD11c^+^CD11b^+^ dendritic cells (DCs) was observed in the lungs of *PTX3^-/-^* mice compared to wild type littermate. Further analysis showed that a short-term OVA exposure led to an increased number of bone marrow common myeloid progenitors (CMP) population concomitantly with increased Ly6C^high^ CCR2^high^ monocytes and CD11c^+^CD11b^+^ DCs in the lungs. Also, pulmonary CD11c^+^CD11b^+^ DCs from OVA-exposed *PTX3^-/-^* mice exhibited enhanced expression of maturation markers, chemokines receptors CCR2, and increased OVA uptake and processing compared to wild type controls. Taken together, our data suggest that PTX3 deficiency heightened lung CD11c^+^CD11b^+^DC numbers and function, hence exacerbating airway inflammatory response.

## Introduction

Allergic asthma is a chronic disease of the airways that is characterized by a maladaptive T helper immune response. Several previously published reports have elaborated on dendritic cells’ crucial role (DCs) in regulating specific helper T cell phenotype and functions in the lungs in response to allergens. Upon allergen/antigen exposure, bone marrow releases CCR2^high^ monocytes that circulate through the blood, reach the pulmonary tissue, and contribute to the generation of inflammatory monocytes derived CD11c+CD11b+DCs (1).

Airway DCs constitute a network with extended dendrites in the epithelium to capture inhaled antigen or allergen then migrate to the T cell zone in draining lymph nodes (2). During this process, immature DCs undergo maturation characterized by upregulation of cell surface expression of MHCII, CD40, CD86 and CD80. DCs process aeroallergens and present allergen peptides to the naïve CD4 T cells that initiate differentiation of naïve CD4 T cells to specific Th cells (3). Upon repeated exposures of allergen, differentiated Th cells return to the effector site in the lungs and initiate inflammation (4). During this stage, mature DCs co-localize with effector T cells in inflammatory areas (1). Deletion of DCs at this stage resulted in ablation of Th2-dependent inflammatory responses in mice, suggesting a critical role of such DCs in regulating effector helper T cell-mediated responses (1).

Pentraxin 3 is a member of a long pentraxin family of pattern recognition receptors that plays a critical role in tuning inflammation in several pulmonary diseases (5). Several reports have shown the production of PTX3 by structural and inflammatory cells in mice and humans (6). Dendritic cells, particularly myeloid DCs but not plasmacytoid DCs, are among the primary producer of PTX3 (5, 6). Furthermore, stimulation with TLR ligands, CD40L, IL-10 and IL-1β induce PTX3 production by DCs (7, 8). PTX3 binds to the late apoptotic T cells and inhibits their internalization by human dendritic cells (9).

Exogenous administration of PTX3 during the early stage of aspergillosis in *p47phox^-/-^* mice restored anti-aspergillus resistance (10). PTX3 treatment of DCs down-regulated production of IL-23 that resulted in a restricted expansion of IL-23-dependent IL-17A producing γδ T cells (10). We recently showed that PTX3 deficiency resulted in an IL-17A dominant inflammation in the mouse model of allergic inflammation (11). CD4 T cells exhibited enhanced IL-17A production in ovalbumin (OVA)-exposed *PTX3^-/-^* mice than their wild-type controls. CD11c^+^CD11b^+^ DCs from *PTX3-/-* mice displayed increased production of Th17 polarizing cytokines, including IL-6 and IL-23, favoring Th17 CD4 T cells (11). However, the mechanism governing the PTX3 effect on regulating dendritic cells in allergic inflammation is not fully understood.

In light of our previous findings (11), we sought to understand the role of PTX3 deletion in shaping CD11c+CD11b+ DCs function in the context of allergic inflammation. Our study identified that OVA exposure induced increased infiltration of CD11c+CD11b+ DCs in the lungs in *PTX3^-/-^* mice compared to wild-type littermate. Upon OVA challenge, *PTX3-/-* bone marrow showed increased frequency of common myeloid progenitors (CMP) population that corresponded with increased Ly6C CCR2^high^ monocytes and CD11c+CD11b+ DCs in the lungs. In addition, pulmonary CD11c+CD11b+ DCs exhibited enhanced expression of maturation markers upon OVA-exposed *PTX3^-/-^* mice than their wild-type controls. These cells also exhibited increased OVA uptake and processing. Together, our data suggest a differential phenotype and function of CD11c+CD11b+ DCs in the absence of PTX3.

## Material and Methods

### Mice

*PTX3^-/-^* and littermate *PTX3 ^+/+^* mice (129SvEv/Bl/6 background) were kindly provided by Dr. M. Matzuk (Baylor College of Medicine, Texas, USA). Animals were housed in individualized ventilated cages with wooden chip bedding in enriched environment conditions in the animal care facility, the University of Manitoba. Animals were maintained in 12 hours dark-light cycle and supplied with standard chow and water *ad libitum*. Animal usage was strictly according to instructed guidelines by the Canadian Council for Animal Care and the University of Manitoba Animal Ethics Board (Protocol Number 15802).

### OVA Sensitization and Challenge, and OVA Uptake and Processing

Six to eight weeks old females *PTX3^-/-^* and *PTX3^+/+^* mice were sensitized by intraperitoneal (IP) injection of OVA (Grade V, Sigma Aldrich Canada) and alum (Imject Alum, Biotechnology). In brief, on day 1, mice were sensitized with IP injection of OVA/alum mixture, 250μl of 8mg/ml alum mixed with 250μl of 4μg/ml OVA dissolved in PBS. On day 11, mice were boosted with IP injection of OVA/alum mixture, as in day 1, and 50μl of OVA (1mg/ml) administered intranasally (IN). On days 19 and 20, *PTX3^-/-^* and *PTX3^+/+^* mice were challenged with IN administration of 50μg of OVA in 50μl PBS solution. 48 hrs after the last challenge, mice were sacrificed, and tissues were harvested. Naïve mice that received PBS only according to the same protocol were used as controls.

To examine *in vivo* OVA uptake, OVA-sensitized mice, as described above, then were challenged intranasally with 50 μg OVA-DQ (Life technology-Invitrogen, Carlsbad, CA) in 50μl of PBS at day 19. At the indicated time, the lung tissue and mediastinal lymph nodes (MLN), were harvested to examine the uptake and processing of DQ-OVA by DCs using flow cytometry.

In another protocol, mice were IN challenged with 50μl of OVA (2mg/ml). Mice were then sacrificed at 12 or 24hrs, and lung tissue, MLN, blood and bone marrow cells were harvested. Again, naïve mice challenged with PBS were used as controls.

### Bone Marrow Single-Cell Preparation

Naïve and OVA exposed *PTX3^+/+^* and *PTX3^-/-^* mice were sacrificed, and tibia and femur bones were collected as described previously (12). Bones were flushed with 10 ml of complete media, bone marrow cells were filtered using a cell strainer, collected cells were centrifuged at 1200 rpm, and the pellet was resuspended in ACK buffer for 5 minutes to lyse red blood cells. ACK was neutralized with complete media, and cells were then centrifuged and resuspended in fresh complete cell culture media before FACS staining.

#### Flow Cytometric Analysis of Pulmonary Dendritic Cells

Pulmonary conventional DC subsets were analyzed by FACS from *PTX3^-/-^* or *PTX3* ^+/+^mice after OVA exposure. Briefly, lungs were removed from mice, and enzymatically digested using 1 mg/ml collagenase IV and 0.5 mg/ml DNase in RPMI 1640 medium, as described previously (13, 14). After Fc blocking, single-cell suspension was incubated with appropriate antibodies (see **Table 1**) followed by acquisition using a BD FACS Canto-II (BD, San Diego, CA) and analyzed using FlowJo software. Similarly, MLN, bone marrow or blood cells were blocked with Fc blocker and stained with respective antibodies (**Table 1**).

**Table 1:** Antibodies used in flow cytometry.

Antibody	Clone	Company
Lin - PE	mix	Biolegend, Inc
SCA-1 - FITC	D7	Biolegend, Inc
CD16/32 - PE-Cy7	93	eBiosciences Inc
CD34 - PB	RAM34	eBiosciences Inc
cKit - APC	2B8	eBiosciences Inc
Ly6C - FITC	HK1.4	eBiosciences Inc
CCR2 - APC	SA203G11	Biolegend, Inc
CCR5 - APC	HM-CCR5	eBiosciences Inc
CCR6 - PE-Cy7	29-2L17	Biolegend, Inc
CD40 - APC	1C10	eBiosciences Inc
MHC II - PB	M5/114.15.2	eBiosciences Inc
CD80 - PE	16-10A1	eBiosciences Inc
CD86 –APC or PE-Cy7	GL-1	eBiosciences Inc
CD45 - PB	30-F11	eBiosciences Inc
B220 - PB	RA3-6B2	ebiosciences Inc
CX3CR1 - BV711	SA011F11	Biolegend, Inc
CD11b - PE-Cy7 or BV711	M1/70	ebiosciences Inc
CD11c - FITC	N418	eBioscience, Inc
Gr-1 - PE	RB6-8C5	Biolegend, Inc
F4/80 - PE	BM8	eBioscience, Inc
Siglec-F - PE	E50-2440	BD Biosciences.

For detecting CMP, bone marrow single cells preparation was labeled with anti-mouse lineage Abs (lin-PE: anti-mouse CD3; anti-mouse Ly-6G/Ly-6C; anti-mouse CD11b; anti-mouse CD45R/B220; anti-mouse TER-119), anti-Ly-6A/E- (Sca1) FITC, anti c-kit-APC, anti-CD16/32 PE-Cy7 and anti-CD34 pacific blue. Labeled cells were then acquired on FACS Canto II (BD Biosciences) and analyzed using FlowJo software.

### Survival Assay

Apoptosis/survival of lung DCs were determined by staining lung cell suspension with annexin V and DAPI using Apoptosis Detection kit FITC according to manufacturer’s instructions (eBioscience Inc.) as we described previously (11). Briefly, cells were first stained with specific fluorochrome-conjugated antibodies, washed with flow buffer once and then in 1X annexin V binding buffer. Resuspended cells were then centrifuged at 1200rpm and stained with FITC labelled annexin V. These cells were incubated at room temperature for 10-15 minutes, followed by washing in 1X binding buffer. After washing, cells were resuspended in 100-200μl 1X binding buffer, to which 1μl of DAPI was added, and incubated at room temperature for at least 5 minutes. Cells were then acquired on FACS Canto II (BD Biosciences) and analyzed using FlowJo software.

### Statistical Analysis

Results are shown as mean-/+ SEM, analyzed using two-way ANOVA (Graph Pad prism 8) and *post hoc* Tukey’s multiple comparisons, and the Mann-Whitney test. Unless otherwise stated, data are collected from at least three experiments; each experiment contained at least three-five mice in each experimental group. p-value <0.05 was considered significant in all quantitative experiments.

## Results

### PTX3-/- Mice Exhibited Enhanced Accumulation of CD11c+CD11b+ DCs in the Lungs Upon OVA Exposure

**Figure 1:**
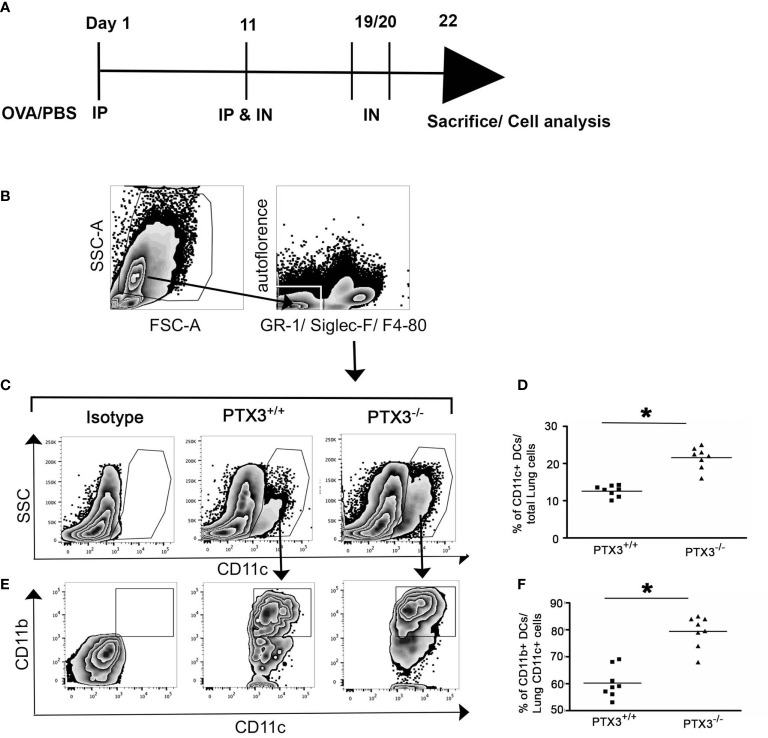
PTX3 deletion results in increased accumulation of CD11c+CD11b+ DCs in the lungs in response to OVA sensitization and challenge. **(A)** Schematic diagram showing the protocol of OVA sensitization and challenge. **(B)** Lung Cells were gated for autofluorescence- Gr-1^-^Siglec F^-^F4/80^-^ CD11c^+^ cells **(C, D)** that were then gated for CD11b+ cells **(E, F)**. Quantification and statistical analysis of FACS data are shown as graphs. n = 6-8/group, *p < 0.01 using Mann-Whitney test.

Recently we showed that the deletion of PTX3 resulted in excessive airway inflammation upon OVA exposure, mainly characterized by enhanced airway and lung tissue infiltration by eosinophils and neutrophils (11). OVA-induced IL-17-dominant inflammation in *PTX3^-/-^* mice compared to their *PTX3* ^+/+^ counterparts was attributed partly to a preponderance of IL-6 and IL-23 producing DCs in *PTX3^-/-^* mice (11). However, whether PTX3 deletion leads to enhanced recruitment of particular DC subsets in the lung upon OVA challenge is unknown. Using the OVA protocol described in **Figure 1A**, we found that *PTX3^-/-^* mice showed enhanced accumulation of CD11c^+^ DCs in the lungs compared to *PTX3* ***^+/+^*** mice upon OVA sensitization and challenge (**Figures 1B–D**). CD11c^+^ DCs are further characterized as proinflammatory CD11b^+/high^ pulmonary DCs known to transport antigen from lungs to the lymphoid organs (15, 16). In *PTX3^-/-^* mice, we observed a concomitant increase in lung CD11c^+^CD11b^+^ DCs upon OVA exposure compared to their WT littermate (**Figures 1E, F**).

Next, we were interested in understanding the plausible mechanism that supported an increased abundance of lung DCs in *PTX3^-/-^* mice. *PTX3^-/-^* CD4 T cells showed enhanced survival compared to CD4 T cells from *PTX3* ***^+/+^*** littermate (11). Thus, we next examined the survival of *PTX3^-/-^* and *PTX3* ***^+/+^*** lung DCs *ex vivo* by staining cells with annexin V and DAPI to assess whether a similar phenomenon existed for DCs in *PTX3^-/-^* mice. Interestingly, OVA–exposed *PTX3* ***^+/+^*** and *PTX3^-/-^* lung DCs showed comparable survival (**Supplementary Figure 1**), suggesting that the survival pathway has no role in increased abundance of DCs in the lungs *PTX3^-/-^* mice.

### PTX3^-/-^ Mice Showed Enhanced Common Myeloid Progenitors Generation in Bone Marrow

**Figure 2:**
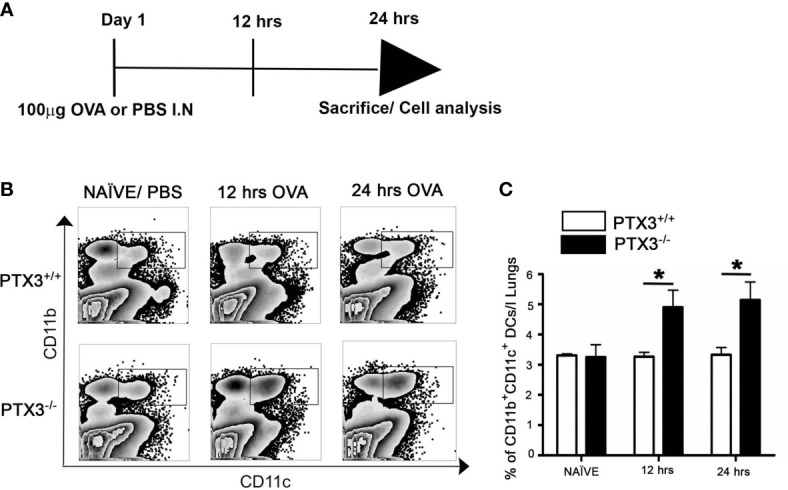
PTX3^-/-^ mice showed increased accumulation of DCs in lungs after short term OVA exposure. **(A)** Schematic diagram showing the protocol of OVA exposure and collection of samples at 12 hrs and 24 hrs. **(B)** Lung Cells were gated for autofluorescence- Gr-1^-^Siglec F^-^F4/80^-^ CD11c^+^ cells. **(C)** Accumulation of lung CD11c^+^ CD11b^+^ DCs 12 hrs and 24 hrs post i.n OVA exposure was compared with naïve lung DCs. **(C)** Quantification and statistical analysis of Flow cytometry data is shown as graphs. n = 6-8/group, *p < 0.01 using two-way ANOVA.

Previously published reports indicated that common myeloid progenitors (CMP) in the bone marrow efficiently give rise to Ly-6C^high^CD11b^+^ inflammatory monocytes (17). Inflammatory monocytes emigrate from the bone marrow, circulate through the blood and rapidly differentiate into CD11c^+^CD11b^+^ DCs in the peripheral tissue under inflammatory conditions (18). Therefore, we aimed to understand whether augmented pulmonary accumulation of CD11b^+^DCs in *PTX3^-/-^* mice was due to enhanced generation of CMPs, increased recruitment of Ly-6C^high^CD11b^+^ monocytes, or differentiation of the latter into inflammatory DCs in the lungs. *PTX3* ***^+/+^*** and *PTX3^-/-^* mice were challenged intranasally with 100μg OVA (**Figure 2A**), and the frequency of CD11b+CD11c+ DCs in the lungs 12 hrs and 24 hrs post-OVA challenge were assessed. As observed in the three weeks OVA sensitization and challenge protocol, short-term OVA challenge increased accumulation of lung CD11c+CD11b^+^DCs in *PTX3^-/-^* mice compared to *PTX3+/+* mice (**Figures 2B, C**, n=6-8, P< 0.01).

**Figure 3:**
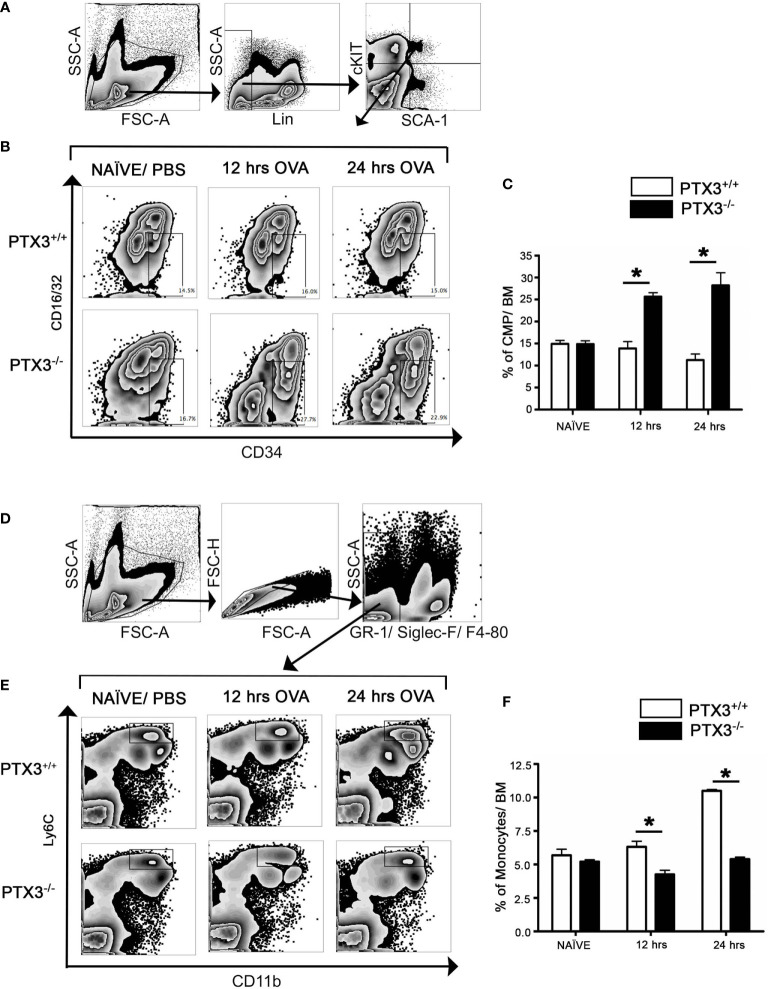
OVA exposure results in increased CMP progenitors and in increased egress of monocytes from the bone marrow in PTX3^-/-^ mice. Lin^-^cKit^+^Sca-1^-^ cells were gated for CMP as **(A)**. **(B, C)** CMP cells from naïve, 12 hrs and 24 hrs post i.n OVA exposed PTX3^-/-^ mice were compared with CMP cells from PTX3^+/+^ mice. **(D)** Gating strategy for Ly6C+ CD11b+ monocytes in bone marrow. **(E, F)** BM monocytes from naïve, 12 hrs and 24 hrs post i.n OVA exposed PTX3^-/-^ mice were compared with monocytes from PTX3^+/+^ mice. Quantification and statistical analysis of Flow cytometry data is shown as graphs. n = 6/group, *p < 0.01 using two-way ANOVA.

We then determined how the OVA challenge affected monocytic lineage progenitors in bone marrow in the presence and absence of PTX3. In contrast to *PTX3^+/+^*, CMP cell count in the bone marrow of *PTX3^-/-^* mice, gated on Lin- cKit+Sca1+ (**Figure 3A**), showed a significant increase upon OVA exposure (**Figures 3B, C**). Conversely, we observed a slight reduction of bone marrow monocytes frequency, gated on GR-F4/80-Siglec- (**Figure 3D**), in *PTX3^-/-^* mice 12 hrs post OVA challenge (**Figures 3E, F**). After 24 hrs of OVA exposure, monocytes frequency in *PTX3^-/-^* mice come back to the baseline level; however, monocytes accumulated in the BM in *PTX3^+/+^* mice (**Figure 3F**).

**Figure 4:**
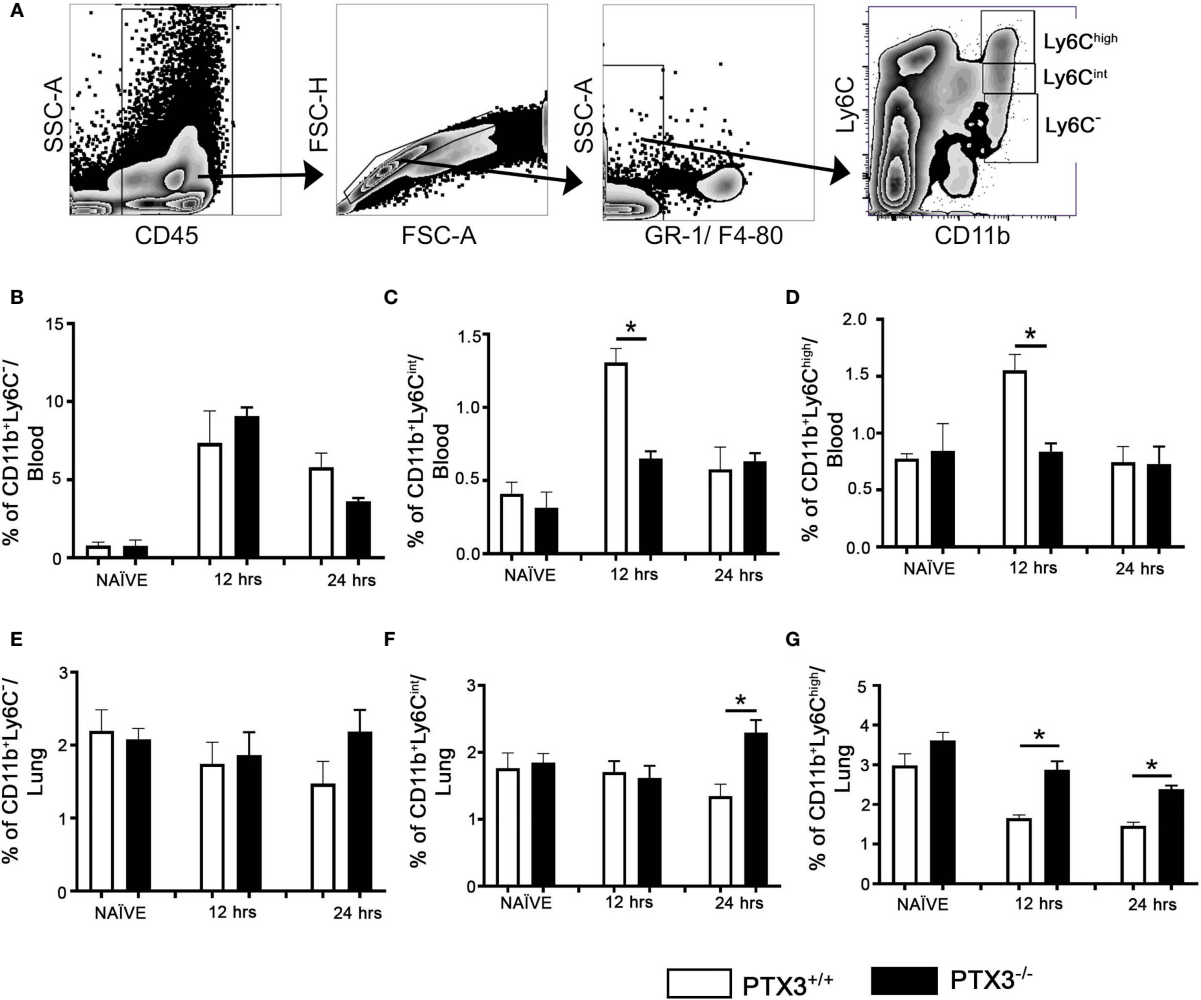
PTX3-/- mice showed increased egress of monocytes from the Blood and increased accumulation of monocytes in the lungs upon OVA exposure. **(A)** Blood monocytes were gated on CD45^+^ Gr1^-^ F4/80 ^-^. Monocytes from blood **(B–D)** and the lungs **(E–G)** at naïve, 12 hrs and 24 hrs post i.n OVA exposed PTX3-/- mice were compared with monocytes from PTX3+/+ mice. Quantification and statistical analysis of Flow cytometry data is shown as graphs. n = 6/group, *p < 0.01 using two-way ANOVA.

To investigate the possibility of BM increased exit to the blood and attraction toward lung tissue in *PTX3 ^-/-^* mice compared to WT mice, we analyzed their frequency within the blood and lung (gated on CD11b+ and Ly6C levels, **Figure 4A**). At 12hrs upon OVA challenge, the Ly6C^int^ CD11b+ blood monocytes frequency increased compared to the baseline in *PTX3^-/-^* mice but was significantly lower than WT mice (**Figure 4C**). Furthermore, the frequency of blood and lung CD11b+ Ly6C^low^ cells was similar between both strains (**Figures 4B, E**). Conversely, at 24h post OVA challenge, the Ly6C^int^ CD11b+lung monocytes frequency was higher in *PTX3^-/-^* compared to WT mice, but no difference was observed in WT mice (**Figure 4F**). Interestingly, in contrast to WT mice, no difference on the frequency of Ly6C^high^ CD11b+ blood monocytes was detected in the blood of *PTX3^-/-^* mice compared to the baseline at 12 and 24hrs post OVA challenge (**Figure 4D**). On the other hand, compared to the baseline level, the frequency of Ly6C^high^ CD11b+ lung monocytes decreased in both *PTX3^-/-^* and WT mice (**Figure 4G**).

### PTX3-/- Lung DCs Exhibited Increased Expression of CCR2, CCR5 and CCR6

**Figure 5:**
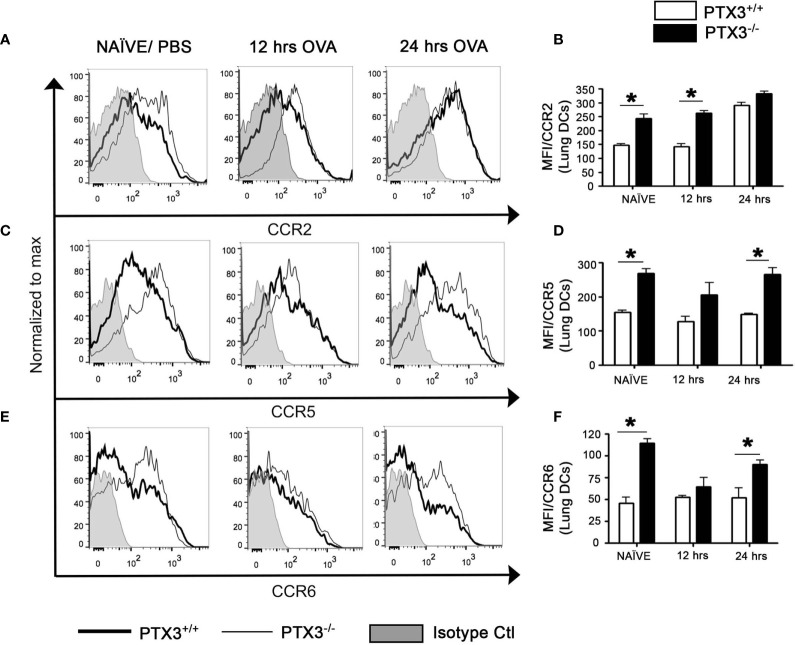
PTX3 deletion affect CCR2, CCR5 and CCR6 expression on lungs DCs. Expression of CCR2 **(A, B)**, CCR5 **(C, D)** and CCR6 **(E, F)** were assessed by flow cytometry on CD11c+CD11b+ DCs from the lungs. Gating strategy as shown in **Figure 1B**. White bars represent PTX3+/+ and black bars represent PTX3^-/-^. Quantification and statistical analysis of Flow cytometry data is shown as graphs. n = 6/group, *p < 0.01 using two-way ANOVA.

CCR2, a chemokine receptor, plays a key role in directing DC precursors to egress from bone marrow to blood, enabling their migration to the tissue sites upon antigen exposure (19). Furthermore, CCR2 is highly expressed by Ly-6C^high^CD11b^+^ inflammatory monocytes compared to Ly-6C^low/int^CD11b^+^ patrolling monocytes (20), and is associated with increased differentiation of Ly-6C^high^CD11b^+^ monocytes into pulmonary CD11c^+^CD11b^+^ DCs (21). Therefore, we investigated the regulation of CCR2 expression in OVA- challenged *PTX3 ^-/-^* mice. Lung DCs from *PTX3^-/-^* showed increased expression of CCR2 at naïve state and 12 hrs post OVA exposure in comparison with WT mice (**Figures 5A, B**) but not at 24 hrs post OVA challenge.

CCR5 is expressed by immature DCs that facilitate migration later to the tissue sites. Immature DCs also express CCR6 that performs a similar function as CCR5. We evaluated the expression of both chemokine receptors on lung DCs and found that the expression of CCR5 (**Figures 5C, D**, P<0.01) and CCR6 (**Figures 5E, F**, P<0.01) was more significant on *PTX3^-/-^* DCs at naïve state as compared to *PTX3^+/+^* DCs. *PTX3^-/-^* lung DCs, although it exhibited increased surface expression of both CCR5 and CCR6 24 hrs post OVA challenge compared to *PTX3^+/+^* lung DCs (**Figures 5D, F**), this expression was not different compared to the baseline expression for CCR5 or even decrease for CCR6 (**Figures 5C–F** and **Supplementary Figure 2**). No apparent difference in their expression was found on monocytes from BM, blood and the lungs (data not shown). Altogether our data demonstrated an inherent increased expression of CCR2, CCR5 and CCR6 on lung DCs *PTX3^-/-^* mice at the baseline and enhanced CCR2 expression upon OVA challenge compared to WT mice.

### PTX3^-/-^ Lung DCs Showed Reduced MHCII but Enhanced CD86 Surface Expression

**Figure 6:**
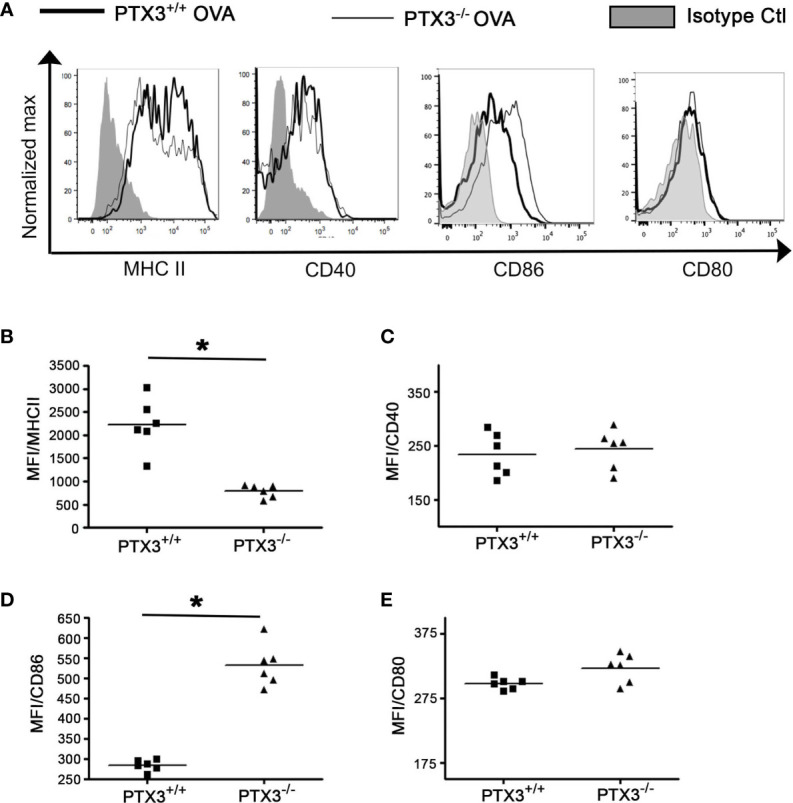
PTX3 deletion results in reduced surface expression of MHCII but an enhanced surface expression of CD80 and CD86 in response to OVA. After 3 weeks of OVA sensitization and challenge regimen, lung CD11b+CD11c+ DCs were analyzed for the expression of MHCII **(A, B)**, CD40 **(A, C)**, CD86 **(A, D)** and CD80 **(A, E)** by flow cytometry. Quantification and statistical analysis of Flow cytometry data is shown as graphs. n = 6 mice group, *p < 0.01 using Mann-Whitney test.

In OVA-exposed *PTX3^-/-^* mice, CD4 T cells exhibited enhanced activation and IL-17A dominant cytokine production (11). Given that DCs process and present antigens to T cells and regulate T cell-dependent inflammatory response through the expression of costimulatory molecules, such as MHCII, CD80, CD86 and CD40, we next assessed whether PTX3 deletion resulted in differential maturation status of DCs, which may explain the phenotype and functions of CD4 T cells. PTX3^-/-^ lung DCs showed lower surface expression of MHC II (**Figures 6A, B**) but an enhanced expression of CD86 (**Figures 6A, D**) upon OVA sensitization/challenge (**Figure 1A**). Surface expression of CD40 (**Figures 6A, C**) and CD80 (**Figures 6A, E**) remained similar in both genotypes at this time point.

We also determined the surface expression of MHCII, CD80, CD86 and CD40 at naïve state and after short-term OVA exposure (**Figure 2A**). Their surface expression was comparable on naïve lung CD11c^+^CD11b^+^ DCs in both *PTX3^-/-^* and WT mice (**Supplementary Figures 3A–D**). Interestingly, lung DCs from *PTX3^-/-^* mice showed an increase in the expression of MHCII, CD80 and CD86 24 hrs OVA challenge compared to WT mice (**Supplementary Figures 3A, B, D**). CD40 surface expression was similar in DCs isolated from OVA challenged (12 hrs and 24 hrs) mice of both genotypes (**Supplementary Figure 3C**).

### PTX3^+/+^ Lung CD11c^+^CD11b^+^ DCs Exhibit Enhanced OVA Uptake and Processing as Compared to PTX3^+/+^ DCs

**Figure 7:**
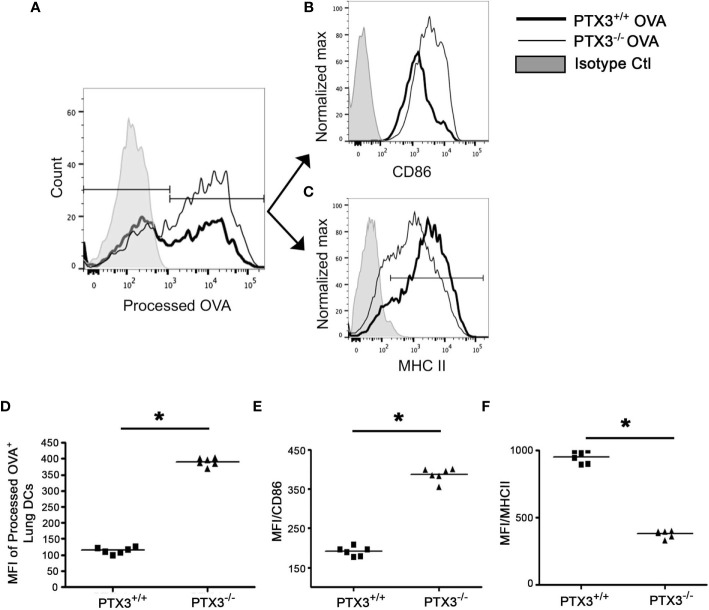
PTX3^-/-^ lung DCs showed enhanced OVA uptake and processing. DQ-OVA was administered intranasally to PTX3^-/-^ and PTX3^+/+^ mice and lung DCs were assessed for processed OVA signal (FITC) 16 hrs post administration **(A, D)**. Surface expression of CD86 **(B, E)** and MHCII **(C, F)** was determined on processed OVA+ lung DCs (gated on CD11b+CD11c+). Quantification and statistical analysis of Flow cytometry data is shown as graphs **(D–F)**. n = 6 mice per group, *p < 0.01 using Mann-Whitney test.

DCs take up allergen/antigen and process them into immunogenic peptides for subsequent T cells presentation (1). To study whether PTX3 deletion affected OVA uptake and presentation by lung DCs, we used DQ-OVA *in vivo*. DQ-OVA is digested proteolytically to release photostable and pH-insensitive green fluorescent peptides by antigen-presenting cells (22). The frequency of DCs, which processed OVA, was higher in *PTX3^-/-^* mice lungs compared to WT mice (**Figures 7A, D**). Similar to total lung DCs, a subset of CD11c^+^CD11b^+^ DCs that were actively processing OVA showed reduced MHC II (**Figures 7C, F**) and increased CD86 (**Figures 7B, E**) surface expression in *PTX3^-/-^* mice.

Lung DCs transport allergen to mediastinal lymph nodes and present allergen peptides to T cells to generate a specific effector T cell response (23). Therefore we explored the migration of DQ-OVA containing lung DCs to mesenteric lymph node (MLN). *PTX3^-/-^* mice showed greater infiltration of processed DQ-OVA containing DCs into the MLN (**Supplementary Figure 4A**). DCs in MLN also showed reduced MHCII (**Supplementary Figure 4B**); however, CD86 expression remained similar (**Supplementary Figure 4C**).

## Discussion

DCs are strategically located in the epithelium to capture antigens/allergens and orchestrate immune response according to the antigen type (1). They are equipped to process allergens and present them to naïve CD4 T cells (4). Recently, we showed that deletion of PTX3 resulted in enhanced infiltration of inflammatory cells in the airways and the lungs, increased production of IgE and enhanced airway hyperreactivity in response to OVA challenge. Also, we identified that PTX3 deletion favored IL-17A-dominant inflammation in the lungs and the draining lymph nodes that is at least in part due to Th17 CD4 T cells (11). In this study, we identified that OVA exposure induced increased infiltration of CD11c+CD11b+ DCs in the lungs of *PTX3-/-* mice compared to wild-type littermate. This effect is associated with an increased number and frequency of common myeloid progenitors (CMP) population that may explain increased Ly6C CCR2^high^ monocytes in the blood and subsequently CD11c+CD11b+ DCs in the lungs. Significantly, pulmonary CD11c+CD11b+ DCs exhibited enhanced expression of maturation markers upon OVA-exposed *PTX3-/-* mice compared to their wild-type controls and exhibited increased OVA uptake and processing. Taken together, our data suggest that PTX3 deficiency mediated increased inflammatory response in the context of allergic asthma is in part due to enhanced DCs function.

In this study, we first showed an increase in CD11b+CD11c+ DCs infiltration into the lungs of *PTX3^-/-^* mice compared to *PTX3^+/+^* mice upon OVA exposure. Unlike *PTX3^-/-^* CD4 T cells (11), *PTX3^-/-^* DCs did not show any apparent difference in survival compared to *PTX3^+/+^* DCs. A plausible explanation of such an increase in inflammatory CD11c+CD11b+ DCs could be enhanced generation of CMP in the bone marrow, which was observed in PTX3 depleted mice compared to their wild-type counterparts. Manz et al. (17) reported that CMP in bone marrow gives rise to granulocyte-monocyte progenitors (GMP), giving rise to functional DCs *in vitro* and *in vivo.* Although in our study, we did not find any difference in GMP population while CMP count was significantly increased in *PTX3^-/-^* compared to *PTX3^+/+^* mice. Monocytes, an intermediate stage between GMP and tissue DCs, give rise to DCs that infiltrate the tissue upon inflammation. To understand whether the increased accumulation of lungs DCs depended on monocytes’ differentiation into DCs, we monitored monocytes in the bone marrow, blood, and the lungs. Reduction of monocytes in the bone marrow and blood but increase in their count in the lungs of *PTX3^-/-^* mice upon OVA exposure partly explains the increased accumulation of lung CD11c+CD11b+ DCs. Such monocytes, which can differentiate to functional DCs upon inflammation, are known as inflammatory monocytes. These are characterized by high expression of CCR2 in contrast to Ly6C^low/int^ CCR2^low^ patrolling monocytes (24).

CCR2 plays a critical role in facilitating the egress of inflammatory monocytes from the bone marrow to blood and from blood to the lungs in response to allergen challenge (19). In our study, we observed that inflammatory monocytes were CCR2^high^ in both genotypes. As CCR2 expression on monocytes at basal level was comparable in *PTX3^-/-^* and *PTX3^+/+^* mice, it rules out the plausibility of an effect of PTX3 deletion on CCR2 expression in naïve state. Although we did not find a significant difference in the CCR2 expression on BM, blood and lung monocytes, lung DCs showed upregulation of CCR2 expression upon OVA exposure. Such observation could stem from two possibilities. *PTX3^-/-^* lung DCs with increased CCR2 represent monocytes that had increased CCR2, which upon antigen encounter differentiated into DCs; and/or the increase in CCR2 in *PTX3^-/-^* DCs regulates their accumulation in the lungs independent of its role on monocytes. Previously, diesel particles induced inflammation was shown to upregulate the expression of CCR2 in the lungs (25). Recruitment of inflammatory monocytes, monocyte-derived DCs and induction of subsequent pulmonary inflammation was also CCR2 dependent (25). Similarly, CCR2-dependent recruitment of monocytes-derived DCs has also been reported upon influenza infection, exposure to LPS and particulate antigens (19, 26). Loss of CCR2 could not be compensated by other chemokines- chemokine receptor systems (19).

Other chemokine receptors, including CCR5 and CCR6, have also been involved in directing monocytes to the tissue sites. These chemokine receptors are expressed on immature DCs and regulate their immigration to the tissue sites (27, 28). Previously Norata et al. (8) also showed enhanced CCR5 expression in the vascular wall of *PTX3^-/-^ apoE^-/-^* mice compared to their *PTX3^+/+^* counterpart. In our study, we observed an increased expression of CCR5 and CCR6 on naïve supposedly immature lung DCs in *PTX3^-/-^* mice at steady sate. Surface expression of CCR5 and CCR6 downregulate as DCs undergo maturation. Similarly, we found a reduction in CCR6 but not CCR5 expression 12 hrs post OVA challenge for *PTX3 -/-* mice, suggesting the possibility of acquiring naïve like phenotype by lung DCs 24 hrs after the challenge. Altogether it seems likely that increased CCR6 on naïve *PTX3^-/-^* lung DCs renders them more responsive to chemokines, which are released immediately after exposure to OVA. CCR6, on the other hand, regulates the migration of Tregs and Th17 cells into the inflammatory region (29). CCR5 expression, known to be associated with Th1 cells (30), was not different in both PTX3 and WT mice lung DCs. Whether the altered expression of these chemokine receptors on lung DCs is also involved in the regulation of Th17 promoting cytokines needs further studies.

PTX3 interacts with P-selectin and inhibits the migration of neutrophils to the peripheral tissues (31). This phenomenon was reversed upon disruption of PTX3 and P-selectin interaction either through PTX3 deletion or antibody-mediated inhibition (32). Furthermore, increased expression of P-selectin has been observed in *PTX3^-/-^* mice, further supporting the likelihood of P-selectin-dependent migration of inflammatory cells in the absence of PTX3. As monocytes are also dependent on P-selectin for migration, it is tempting to hypothesize this pathway’s involvement in enhanced monocytes and CD11c+CD11b+ DCs accumulation in the lungs.

In the lungs, DCs exist in an immature state and initiate their maturation upon allergen exposure process. Mature DCs process allergen, present allergen epitopes through MHCII along with the expression of an array of costimulatory molecules including CD40, CD80, and CD86, which are well known as maturation markers of DCs. *PTX3^-/-^* CD11c+CD11b+ DCs showed a greater ability to uptake and process OVA compared to *PTX3^+/+^* DCs. Surprisingly, *PTX3^-/-^* DCs expressed a reduced level of MHCII. Whether the lower expression of MHCII by *PTX3 ^-/-^* DCs was due to increased recycling of MHCII or reduced biosynthesis or transport to the membrane is unknown (33). Engagement of CD80/86 by CD28 induces the production of IL-6 by DCs (34). In our work, we observed enhanced expression of CD80 and CD86 by *PTX3^-/-^* CD11c+CD11b+ DCs in contrast to *PTX3^+/+^* CD11c+CD11b+ DCs, which is also concurrent with increased production of IL-6 by *PTX3^-/-^* DCs (11). Consistent with increased maturation, antigen uptake, and processing, we also observed more significant migration of OVA peptide containing CD11c+CD11b+ DCs from lungs to the draining lymph nodes in *PTX3^-/-^* mice as compared to their wild type controls. Altogether our data provide plausible insight into how deletion of PTX3 has rendered pulmonary DCs hyper mature upon OVA exposure and affected their function.

## Data Availability Statement

The raw data supporting the conclusions of this article will be made available by the authors, without undue reservation.

## Ethics Statement

Animal usage was strictly according to instructed guidelines by the Canadian Council for Animal Care and the University of Manitoba Animal Ethics Board (Protocol Number 15802).

## Author Contributions

AG supervised the study, provided scientific insight, reviewed and edited the manuscript. JB performed the animal study and analyzed the data, and drafted the manuscript. LK analyzed the data, drafted and revised the manuscript. LS performed the animal study and analyzed the data. AM helped in the animal study and the chemokines experiments. LB provided insight into the study design and manuscript writing. All authors contributed to the article and approved the submitted version.

## Funding

This study was funded by a Canadian Institute of Heath Research bridge fund (Project#149062).

## Conflict of Interest

The authors declare that the research was conducted in the absence of any commercial or financial relationships that could be construed as a potential conflict of interest.

## Supplementary Material

Supplementary Figure 1 PTX3+/+ and PTX3-/- lung DCs show comparable survival. **(A)** Survival of lung DCs (CD11c+CD11b+) was assessed by annexin V and DAPI staining. % of gated cells is presented as mean+/- SEM in graph **(B)**, n = 8-10/group. *P < 0.01.

Supplementary Figure 2 PTX3 deletion affect CCR2 expression on lungs DCs. Expression of CCR2 **(A)**, CCR5 **(B)** and CCR6 **(C)** were assessed by flow cytometry on CD11c+CD11b+ DCs from the lungs. Quantification and statistical analysis of Flow cytometry data was done using two way ANOVA. n = 6/group, p values are indicated above the graph bars.

Supplementary Figure 3 Quantification and statistical analysis of the expression of MHCII **(A)**, CD86 **(B)**, CD40 **(C)** and CD80 **(D)** at naïve state, 12 and 24 hrs after single i.n OVA challenge. n = 6/group, *p < 0.01.

Supplementary Figure 4 PTX3-/- mice exhibited increased accumulation of OVA containing DCs in MLN. **(A)** Sensitized and challenged mice were IN challenged with DQ-OVA at day 19, then 24 hrs post i.n administration of DQ-OVA, PTX3^-/-^ MLN DCs that contained processed OVA were compared with those from PTX3+/+ mice. Surface expression of MHCII **(B)** and CD86 **(C)** was determined on processed OVA+ PTX3^+/+^ and PTX3-/- MLN DCs. Result depicted is a representative of 2 experiments with n = 4 mice per each group.

## References

[1] Lambrecht BN Hammad H. The Immunology of Asthma. Nat Immunol (2015) 16:45–56. 10.1038/ni.3049 25521684

[2] Tibbitt CA Stark JM Martens L Ma J Mold JE Deswarte K. Single-Cell RNA Sequencing of the T Helper Cell Response to House Dust Mites Defines a Distinct Gene Expression Signature in Airway Th2 Cells. Immunity (2019) 51:169–84.e165. 10.1016/j.immuni.2019.05.014 31231035

[3] Butcher MJ Zhu J. Recent Advances in Understanding the Th1/Th2 Effector Choice. Fac Rev (2021) 10:30. 10.12703/r/10-30 33817699 PMC8009194

[4] Schuijs MJ Hammad H Lambrecht BN. Professional and ‘Amateur’ Antigen-Presenting Cells In Type 2 Immunity. Trends Immunol (2019) 40:22–34. 10.1016/j.it.2018.11.001 30502024 PMC7610811

[5] Balhara J Koussih L Zhang J Gounni AS. Pentraxin 3: An Immuno-Regulator in the Lungs. Front Immunol (2013) 4:127. 10.3389/fimmu.2013.00127 23755050 PMC3668324

[6] Bottazzi B Inforzato A Messa M Barbagallo M Magrini E Garlanda C. The Pentraxins PTX3 and SAP in Innate Immunity, Regulation of Inflammation and Tissue Remodelling. J Hepatol (2016) 64:1416–27. 10.1016/j.jhep.2016.02.029 PMC5414834 26921689

[7] Introna M Alles VV Castellano M Picardi G De Gioia L Bottazzai B. Cloning of Mouse Ptx3, A New Member of the Pentraxin Gene Family Expressed at Extrahepatic Sites. Blood (1996) 87:1862–72. 10.1182/blood.V87.5.1862.1862 8634434

[8] Doni A Michela M Bottazzi B Peri G Valentino S Polentarutti N. Regulation of PTX3, a Key Component of Humoral Innate Immunity in Human Dendritic Cells: Stimulation by IL-10 and Inhibition by IFN-Gamma. J Leukoc Biol (2006) 79:797–802. 10.1189/jlb.0905493 16461742

[9] Rovere P Peri G Fazzini F Bottazzi B Doni A Bondanza A. The Long Pentraxin PTX3 Binds to Apoptotic Cells and Regulates Their Clearance by Antigen-Presenting Dendritic Cells. Blood (2000) 96:4300–6. 10.1182/blood.V96.13.4300.h8004300_4300_4306 11110705

[10] D’Angelo C De Luca A Zelante T Bonifazi P Moretti S Giovannini G. Exogenous Pentraxin 3 Restores Antifungal Resistance and Restrains Inflammation in Murine Chronic Granulomatous Disease. J Immunol (2009) 183:4609–18. 10.4049/jimmunol.0900345 19734205

[11] Balhara J Shan L Zhang J Muhuri A Halayko AJ Almiski MS. Pentraxin 3 Deletion Aggravates Allergic Inflammation Through a TH17-Dominant Phenotype and Enhanced CD4 T-Cell Survival. J Allergy Clin Immunol (2017) 139:950–63.e959. 10.1016/j.jaci.2016.04.063 27567326 PMC6317853

[12] Gupta G Mou Z Jia P Sharma R Zayats R Viana SM. The Long Pentraxin 3 (PTX3) Suppresses Immunity to Cutaneous Leishmaniasis by Regulating CD4(+) T Helper Cell Response. Cell Rep (2020) 33:108513. 10.1016/j.celrep.2020.108513 33326783

[13] Movassagh H Shan L Mohammed A Halayko AJ Gounni AS. Semaphorin 3E Deficiency Exacerbates Airway Inflammation, Hyperresponsiveness, and Remodeling in a Mouse Model of Allergic Asthma. J Immunol (2017) 198:1805–14. 10.4049/jimmunol.1601514 28108561

[14] Plantinga M Guilliams M Vanheerswynghels M Deswarte K Branco-Madeira F Toussaint W. Conventional and Monocyte-Derived CD11b(+) Dendritic Cells Initiate and Maintain T Helper 2 Cell-Mediated Immunity to House Dust Mite Allergen. Immunity (2013) 38:322–35. 10.1016/j.immuni.2012.10.016 23352232

[15] Shi C Pamer EG. Monocyte Recruitment During Infection and Inflammation. Nat Rev Immunol (2011) 11:762–74. 10.1038/nri3070 PMC3947780 21984070

[16] Chen K Liu M Liu Y Wang C Yoshimura T Gong W. Signal Relay by CC Chemokine Receptor 2 (CCR2) and Formylpeptide Receptor 2 (Fpr2) in the Recruitment of Monocyte-Derived Dendritic Cells in Allergic Airway Inflammation. J Biol Chem (2013) 288:16262–73. 10.1074/jbc.M113.450635 PMC3675565 23603910

[17] Manz MG Traver D Miyamoto T Weissman IL Akashi K. Dendritic Cell Potentials of Early Lymphoid and Myeloid Progenitors. Blood (2001) 97:3333–41. 10.1182/blood.V97.11.3333 11369621

[18] GeurtsvanKessel CH Lambrecht BN. Division of Labor Between Dendritic Cell Subsets of the Lung. Mucosal Immunol (2008) 1:442–50. 10.1038/mi.2008.39 19079211

[19] Robays LJ Maes T Lebecque S Lira SA Kuziel WA Brusselle GG. Chemokine Receptor CCR2 But Not CCR5 or CCR6 Mediates the Increase in Pulmonary Dendritic Cells During Allergic Airway Inflammation. J Immunol (2007) 178:5305–11. 10.4049/jimmunol.178.8.5305 17404315

[20] Palframan RT Jung S Cheng G Weninger W Luo Y Dorf M. Inflammatory Chemokine Transport and Presentation in HEV: A Remote Control Mechanism for Monocyte Recruitment to Lymph Nodes in Inflamed Tissues. J Exp Med (2001) 194:1361–73. 10.1084/jem.194.9.1361 PMC2195988 11696600

[21] Osterholzer JJ Chen GH Olszewski MA Curtis JL Huffnagle GB Toews GB. Accumulation of CD11b+ Lung Dendritic Cells in Response to Fungal Infection Results From the CCR2-Mediated Recruitment and Differentiation of Ly-6Chigh Monocytes. J Immunol (2009) 183:8044–53. 10.4049/jimmunol.0902823 PMC4043300 19933856

[22] Schmudde I Strover HA Vollbrandt T Konig P Karsten CM Laumonnier Y. C5a Receptor Signalling in Dendritic Cells Controls the Development of Maladaptive Th2 and Th17 Immunity in Experimental Allergic Asthma. Mucosal Immunol (2013) 6:807–25. 10.1038/mi.2012.119 23212198

[23] Trombetta ES Mellman I. Cell Biology of Antigen Processing *In Vitro* and *In Vivo* . Annu Rev Immunol (2005) 23:975–1028. 10.1146/annurev.immunol.22.012703.104538 15771591

[24] Menezes S Melandri D Anselmi G Perchet T Loschko J Dubrot J. The Heterogeneity of Ly6C(hi) Monocytes Controls Their Differentiation Into iNOS(+) Macrophages or Monocyte-Derived Dendritic Cells. Immunity (2016) 45:1205–18. 10.1016/j.immuni.2016.12.001 PMC5196026 28002729

[25] Provoost S Maes T Joos GF Tournoy KG. Monocyte-Derived Dendritic Cell Recruitment and Allergic T(H)2 Responses After Exposure to Diesel Particles are CCR2 Dependent. J Allergy Clin Immunol (2012) 129:483–91. 10.1016/j.jaci.2011.07.051 21906792

[26] Maus UA Wellmann S Hampl C Kuziel WA Srivastava M Mack M. CCR2-Positive Monocytes Recruited to Inflamed Lungs Downregulate Local CCL2 Chemokine Levels. Am J Physiol Lung Cell Mol Physiol (2005) 288:L350–358. 10.1152/ajplung.00061.2004 15516494

[27] Bracke KR D’Hulst A I Maes T Demedts IK Moerloose KB Kuziel WA. Cigarette Smoke-Induced Pulmonary Inflammation, But Not Airway Remodelling, Is Attenuated in Chemokine Receptor 5-Deficient Mice. Clin Exp Allergy (2007) 37:1467–79. 10.1111/j.1365-2222.2007.02808.x 17883726

[28] Bracke KR D’Hulst A I Maes T Moerloose KB Demedts IK Lebecque S. Cigarette Smoke-Induced Pulmonary Inflammation and Emphysema Are Attenuated in CCR6-Deficient Mice. J Immunol (2006) 177:4350–9. 10.4049/jimmunol.177.7.4350 16982869

[29] Yamazaki T Yang XO Chung Y Fukunaga A Nurieva R Pappu B. CCR6 Regulates the Migration of Inflammatory and Regulatory T Cells. J Immunol (2008) 181:8391–401. 10.4049/jimmunol.181.12.8391 PMC2752441 19050256

[30] Odum N Bregenholt S Eriksen KW Skov S Ryder LP Bendtzen K. The CC-Chemokine Receptor 5 (CCR5) Is a Marker of, But Not Essential for the Development of Human Th1 Cells. Tissue Antigens (1999) 54:572–7. 10.1034/j.1399-0039.1999.540606.x 10674971

[31] Deban L Russo RC Sironi M Moalli F Scanziani M Zambelli V. Regulation of Leukocyte Recruitment by the Long Pentraxin PTX3. Nat Immunol (2010) 11:328–34. 10.1038/ni.1854 20208538

[32] Chen J Matzuk MM Zhou XJ Lu CY. Endothelial Pentraxin 3 Contributes to Murine Ischemic Acute Kidney Injury. Kidney Int (2012) 82:1195–207. 10.1038/ki.2012.268 PMC3499641 22895517

[33] Roche PA Furuta K. The Ins and Outs of MHC Class II-Mediated Antigen Processing and Presentation. Nat Rev Immunol (2015) 15:203–16. 10.1038/nri3818 PMC6314495 25720354

[34] Koorella C Nair JR Murray ME Carlson LM Watkins SK Lee KP. Novel Regulation of CD80/CD86-Induced Phosphatidylinositol 3-Kinase Signaling by NOTCH1 Protein in Interleukin-6 and Indoleamine 2,3-Dioxygenase Production by Dendritic Cells. J Biol Chem (2014) 289:7747–62. 10.1074/jbc.M113.519686 PMC3953285 24415757

