# Supplementary figures and images for: PTX3 Deficiency Promotes Enhanced Accumulation and Function of CD11c+CD11b+ DCs in a Murine Model of Allergic Inflammation
Source: Front Immunol. 2021 Jul 9;12:641311. doi: 10.3389/fimmu.2021.641311 (PMC8299994; doi:10.3389/fimmu.2021.641311)

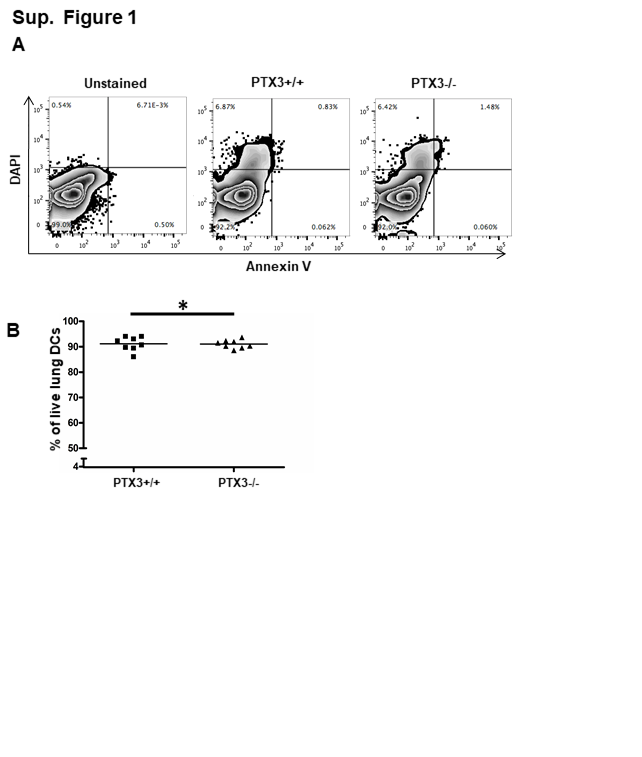

Supplement: Supplementary Figure 1 — PTX3+/+ and PTX3-/- lung DCs show comparable survival. (A) Survival of lung DCs (CD11c+CD11b+) was assessed by annexin V and DAPI staining. % of gated cells is presented as mean+/- SEM in graph (B), n = 8-10/group. *P < 0.01. [file Image_1.tif]

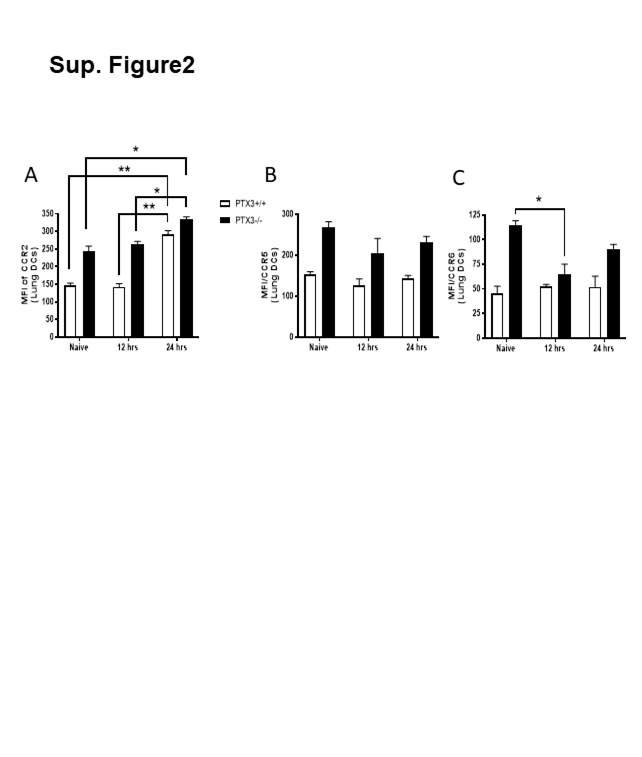

Supplement: Supplementary Figure 2 — PTX3 deletion affect CCR2 expression on lungs DCs. Expression of CCR2 (A), CCR5 (B) and CCR6 (C) were assessed by flow cytometry on CD11c+CD11b+ DCs from the lungs. Quantification and statistical analysis of Flow cytometry data was done using two way ANOVA. n = 6/group, p values are indicated above the graph bars. [file Image_2.tif]

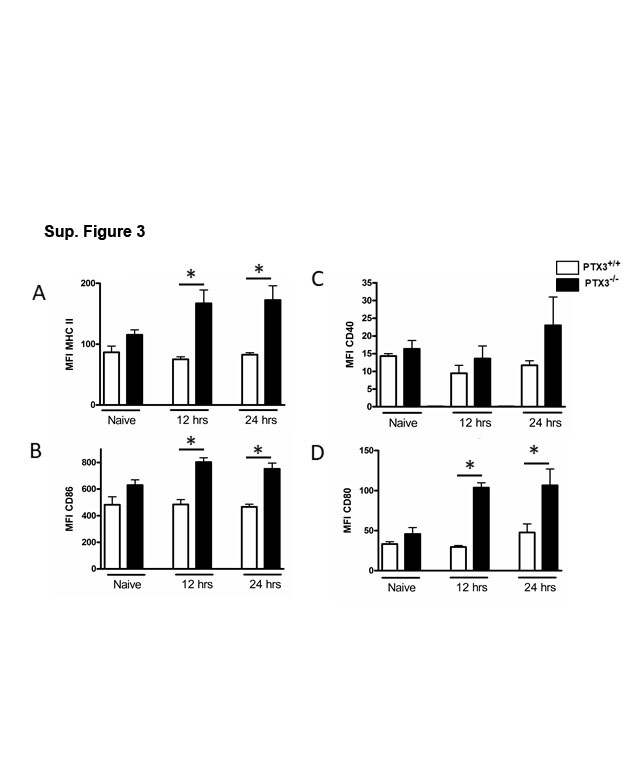

Supplement: Supplementary Figure 3 — Quantification and statistical analysis of the expression of MHCII (A), CD86 (B), CD40 (C) and CD80 (D) at naïve state, 12 and 24 hrs after single i.n OVA challenge. n = 6/group, *p < 0.01. [file Image_3.jpg]

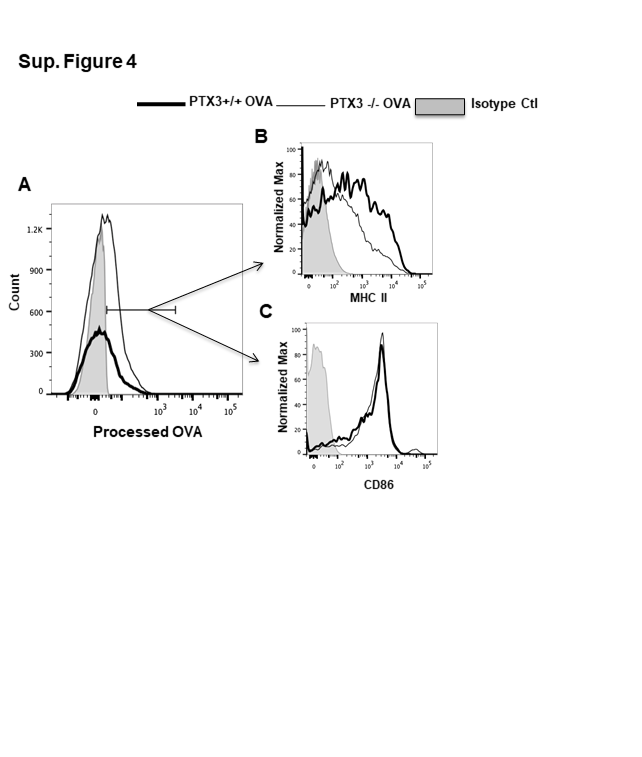

Supplement: Supplementary Figure 4 — PTX3-/- mice exhibited increased accumulation of OVA containing DCs in MLN. (A) Sensitized and challenged mice were IN challenged with DQ-OVA at day 19, then 24 hrs post i.n administration of DQ-OVA, PTX3-/- MLN DCs that contained processed OVA were compared with those from PTX3+/+ mice. Surface expression of MHCII (B) and CD86 (C) was determined on processed OVA+ PTX3+/+ and PTX3-/- MLN DCs. Result depicted is a representative of 2 experiments with n = 4 mice per each group. [file Image_4.tif]
